# Obesity alters the gustatory perception of lipids in the mouse: plausible involvement of lingual CD36[S]

**DOI:** 10.1194/jlr.M039446

**Published:** 2013-09

**Authors:** Michael Chevrot, Arnaud Bernard, Déborah Ancel, Marjorie Buttet, Céline Martin, Souleymane Abdoul-Azize, Jean-François Merlin, Hélène Poirier, Isabelle Niot, Naim Akhtar Khan, Patricia Passilly-Degrace, Philippe Besnard

**Affiliations:** Physiologie de la Nutrition et Toxicologie (NUTox), UMR U866 INSERM/Université de Bourgogne/AgroSup Dijon, F-21000 Dijon, France

**Keywords:** long-chain fatty acids, taste sensitivity, calcium imaging, health

## Abstract

A relationship between orosensory detection of dietary lipids, regulation of fat intake, and body mass index was recently suggested. However, involved mechanisms are poorly understood. Moreover, whether obesity can directly modulate preference for fatty foods remains unknown. To address this question, exploration of the oral lipid sensing system was undertaken in diet-induced obese (DIO) mice. By using a combination of biochemical, physiological, and behavioral approaches, we found that *i*) the attraction for lipids is decreased in obese mice, *ii*) this behavioral change has an orosensory origin, *iii*) it is reversed in calorie-restricted DIO mice, revealing an inverse correlation between fat preference and adipose tissue size, *iv*) obesity suppresses the lipid-mediated downregulation of the lipid-sensor CD36 in circumvallate papillae, usually found during the refeeding of lean mice, and *v*) the CD36-dependent signaling cascade controlling the intracellular calcium levels ([Ca^2+^]_i_) in taste bud cells is decreased in obese mice. Therefore, obesity alters the lipid-sensing system responsible for the oral perception of dietary lipids. This phenomenon seems to take place through a CD36-mediated mechanism, leading to changes in eating behavior.

Existence of an attraction for dietary lipids is observed in both rodents (1–3) and humans (4, 5). Over the last decade, mechanisms leading to this stereotyped behavior have been actively studied, and compelling evidence supports the implication of a taste component in the detection of lipids [i.e., long-chain fatty acids (LCFAs)], in addition to textural, olfactory and postingestive cues. In mice, it was shown that *i*) gene invalidation of two unrelated LCFA receptors specifically expressed in taste buds (i.e., CD36 and GPR120) renders animals unable to properly detect lipids during behavioral tests (6, 7), *ii*) LCFAs trigger a signaling cascade in freshly isolated CD36-positive taste bud cells leading to a rise in [Ca^2+^]_i_ and to a neurotransmitter release (8, 9), and *iii*) the subsequent lipid signal uses peripheral and central nervous circuitry involved in gustation (7, 10). Recent data have highlighted important differences between lingual CD36 and GPR120 suggesting distinct, but complementary, functions in mouse taste buds. In contrast to GPR120, expression of CD36 protein in circumvallate papillae (CVP) is lipid sensitive (11). Indeed, lingual CD36 protein levels displayed a rapid decrease in CVP when fasted mice were refed a diet containing lipids. It was suggested that the lipid-mediated downregulation of CD36 in taste bud cells might modulate the motivation for fat consumption during a meal, initially high and then gradually decreasing secondary to the food intake, a phenomenon reminiscent of sensory-specific satiety (12). For GPR120, an implication in the glucagon-like peptide-1 (GLP-1) release by mouse CVP, producing a modulation of sweet and “fatty” taste sensitivity, was proposed (13).

Psychophysical experiments demonstrate that humans are also able to detect very low concentrations of LCFA in oral cavity under conditions in which textural and olfactory cues are minimized (14). Recent data suggest an implication of lingual CD36 in this phenomenon. Indeed, obese patients with the common single nucleotide polymorphism A/A genotype at rs1761667, known to reduce the CD36 gene expression (15), displayed a higher detection threshold (i.e., low fat perception sensitivity) for lipids than G/G controls (16) and showed a greater attraction for added fats and oils (17). Therefore, genetic predispositions may affect the preference for fat, which might induce changes in eating behavior and obesity risk. Given the obesity epidemic, an important alternative issue is to determine whether obesity itself leads to changes in the orosensory perception of dietary fat. Interestingly, a relationship between orosensory sensitivity for lipids and body mass index has been recently reported in human (18). Moreover, a comparison of obese and nonobese children and adolescents for the detection of sweet, umami, bitter, salty, and sour tastes demonstrates that obesity affects taste sensitivity. Indeed, obese subjects display a low ability to correctly identify the different taste modalities (19). However, mechanisms involved in the modulation of taste sensitivity are poorly understood. To address this question, mice were subjected to nutritional challenges (obesogenic or calorie-restricted diets), and a combination of biochemical, physiological, and behavioral approaches was used to analyze the impact of obesity on fat preference.

## MATERIALS AND METHODS

### Animals and diets

French guidelines for the use and the care of laboratory animals were followed, and experimental protocols were approved by the Animal Ethics Committee of Burgundy University. Six week-old C57Bl/6 mice were purchased from Charles River Laboratories, France. Animals were housed in filtered cages in a controlled environment (constant temperature and humidity, dark period from 7 PM to 7 AM). The mice had free access to tap water during the experiment. After a 1 week acclimatization period, the mice were fed ad libitum either a standard laboratory chow (4RF21, Mucedola, Italy; containing 3% fat, w/w) or two different obesogenic diets: a high-fat (HF) diet, rich in saturated FAs and a high-fat/high-sucrose (HFHS) diet (**Table 1**). Some diet-induced obese (DIO) mice fed the HF diet were calorie restricted (= 60% energy of ad libitum energy intake). Body mass and food consumption were recorded weekly. Body composition (fat mass, lean mass, and total body water) was measured by EchoMRI (Echo Medical Systems; Houston, TX).

**TABLE 1.** Diet composition

		HF		HFHS	
Contents (% w/w)		Control (4RF21 Mucedola)	HF (4RF25 Mucedola + Palm oil)	Control (Research Diet)	HFHS (Research Diet)
Proteins		18.5	15.0	19	26.2
Carbohydrates	Starch	53.5	34.41	63.1	—
	Sucrose	—	—	—	26.1
Fats	Soya oil	3	2.4	6.5	3.2
	Palm	—	31.8	—	—
	Lard	—	—	—	31.7
of which	Saturated FA	0.5	16.7	1	16.3
	Monounsaturated FA	0.5	13.0	1.6	13.4
	Polyunsaturated FA	1.3	4.5	3.9	5.1
Energy	(kcal/100g)	315	505.8	386.9	523.2

### Tissues and blood samples

CVP from control and DIO mice were isolated according to the procedure described elsewhere (6). Briefly, lingual epithelium was separated from connective tissue by enzymatic dissociation (elastase and dispase mixture, 2 mg/ml each in Tyrode buffer: 140 mM NaCl, 5 mM KCl, 10 mM HEPES, 1 mM CaCl_2_, 10 mM glucose, 1 mM MgCl_2_, 10 mM Na pyruvate, pH 7.4), and the papilla was dissected under a binocular microscope. CVP were either used immediately for the calcium imaging assays or snap-frozen in liquid nitrogen then stored at −80°C until CD36 determination by Western blotting. Plasma was obtained after centrifugation of blood (5,000 *g* for 10 min, 4°C). Plasma insulin levels were assayed by using a commercial ELISA kit (Mercodia, Sweden), and plasma glucose and triglyceride levels were determined with enzymatic reaction kits (Biomerieux, France).

### Behavioral experiments

Two different tests, consisting of offering simultaneously a control and an experimental solution (two-bottle preference test) or successively in a randomized manner (licking test) were used.

#### Two-bottle preference tests.

Mice were offered two bottles at the beginning of the dark period for 12 h. Control and experimental bottles contained 0.3% xanthan gum (w/v; Sigma-Aldrich) in water in order to emulsify the oil and to minimize textural cues between the two solutions. Animals were subjected to a choice between control or oily solution containing 0.02%, 0.2%, and 2.0% rapeseed oil (w/v), successively. To avoid the development of side preference, position of each bottle was inversed at each test. At the end of the test, the consumption of each solution was analyzed by weighing the bottles, and the percent of preference for the experimental solution was calculated (ratio consumption of the experimental solution upon total consumption).

#### Licking tests.

This test was performed to analyze the short-term (1–5 min) preference for an LCFA by using computer-controlled lickometers (Med Associates). Animals were successively subjected to the control or the experimental solution, and the number of licks given on each bottle by min was determined. Mice were food and water deprived 6 h before the test, which took place 6 h after the beginning of the dark period. After a training period required to learn the procedure, mice were randomly subjected to a bottle containing a control solution (mineral oil; Cooper, France) or a bottle containing an experimental one [mineral oil + 0.5% oleic acid (OLA); Sigma-Aldrich] for 15 min. Then mice were offered the other bottle for an additional 15 min session. In this experiment, data were analyzed for 1 or 5 min from the first lick to exclude postingestive signals. OLA was chosen because it is the main LCFA found in rapeseed oil.

### Immunohistochemistry

CVP from fasted control and DIO mice were fixed for 3 h in ice-cold 4% paraformaldehyde in 0.1 M phosphate buffer, pH 7.4. Samples were cryoprotected by incubation in 15% sucrose in 0.1 M phosphate buffer for 2 h, followed by overnight treatment with 30% sucrose in phosphate buffer. CVP were then embedded in OCT medium (Tissue-Tek; Sakura Finetek) and stored at −80°C. Cryostat sections (10 µm) were air dried for 45 min at room temperature and rehydrated in 0.1 M PBS (pH 7.4) for 10 min. Rehydrated sections were blocked in 10% FA-free BSA and 0.2% Triton X-100 in PBS for 1 h at room temperature and incubated overnight at 4°C with a polyclonal anti-rabbit CD36 antibody (1:50; Abcam ab80978). After washing, sections were incubated for 3 h at room temperature with a fluorescent anti-rabbit secondary antibody (Alexa 568, 1:200 dilution; Invitrogen) and then counterstained with Hoechst reactive (0.05 mg/ml; Sigma-Aldrich) to stain the nuclei. Slices were analyzed under an epifluorescent microscope (Axiovert 200M). No fluorescent staining was observed when the primary antibody was omitted.

### Western blotting

Freshly isolated mouse CVP were homogenized using a micro-potter in a TSE buffer [50 mM Tris HCl, 150 mM NaCl, 1 mM EDTA, 1% Igepal CA-630, 5 µl/ml anti-protease cocktail (Sigma-Aldrich), and 100 µl/10 ml antiphosphatase (Thermo Fischer)]. Samples were stored on ice for 30 min and then centrifuged (12,000 *g*, 15 min, 4°C). Lysates were used immediately or stored at −80°C until the assay. Protein concentrations in homogenates were assayed using a BCA kit (Thermo Fisher). Denaturated proteins (4 µg) were separated by SDS-PAGE (10%) and transferred to a polyvinylidene difluoride membrane by electroblotting. After being blocked overnight using a TBS buffer containing 5% BSA and 0.05% Tween-20, membranes were incubated for 3 h with an anti-CD36 antibody (1:1,000 dilution; R and D Systems). After a set of washes, the appropriate peroxidase-conjugated secondary antibody was added. Antibody labeling was detected by chemiluminescence (ECL-plus reagent; Perkin Elmer). β-Actin (1:100 dilution; Santa Cruz) was used as an internal reference protein.

Each point corresponds to a pool of total proteins from three mice CVP. Each experiment was repeated three times.

### Measurement of Ca^2+^ signaling

CD36-positive taste buds cells were freshly isolated from mouse CVP as described previously (8, 10) and further cultured for 24 h in Willico-Dish wells containing RPMI-1640 medium, supplemented with 10% fetal calf serum, 200 U/ml penicillin, and 0.2 mg/ml streptomycin. The following day, the CD36-positive taste bud cells were gently washed with a buffer containing the following: 3.5 mM KH_2_PO_4_; 17.02 mM Na_2_HPO_4_; 136 mM NaCl, pH 7.4. The cells were then incubated with Fura-2/AM (1 µM) for 60 min at 37°C in loading buffer that contained the following: 110 mM NaCl; 5.4 mM KCl; 25 mM NaHCO_3_; 0.8 mM MgCl_2_; 0.4 mM KH_2_PO_4_; 20 mM Hepes-Na; 0.33 mM Na_2_HPO_4_; 1.2 mM CaCl_2_, pH 7.4.

After loading, the cells (2 × 10^6^/ml) were washed three times and remained suspended in the identical buffer. The changes in intracellular Ca^2+^ (*F*_340_/*F*_380_) were monitored under the Nikon microscope (TiU) by using an S-fluor 40× oil immersion objective. The planes were taken at *Z* intervals of 0.3 μm, and NIS-Elements software was used to deconvolve the images. The microscope was equipped with an EM-CCD (Lucas) camera for real-time recording of 16 bit digital images. The dual excitation fluorescence imaging system was used for studies of individual cells. The changes in intracellular Ca^2+^ were expressed as ΔRatio, which was calculated as the difference between the peak *F*_340_/*F*_380_ ratio. The data were summarized from the large number of individual cells (20–40 cells in a single run, with three to nine identical experiments done in at least three cell preparations). For experiments in Ca^2+^−free medium, CaCl_2_ was replaced by EGTA (2 mM).

Each point corresponds to a pool of total proteins from 25 mice CVP. Each experiment was repeated three times.

### Statistics

Results are expressed as mean ± SEM. The significance of differences between groups was evaluated with XLSTAT (Addinsoft; France). We first checked that the data for each group were normally distributed and that variances were equal. We then carried out two-tailed Student's *t*-tests, Mann-Whitney tests, or Pearson correlation.

## RESULTS

### Mice fed an HF diet display a lower preference for lipids

**Fig. 1.**
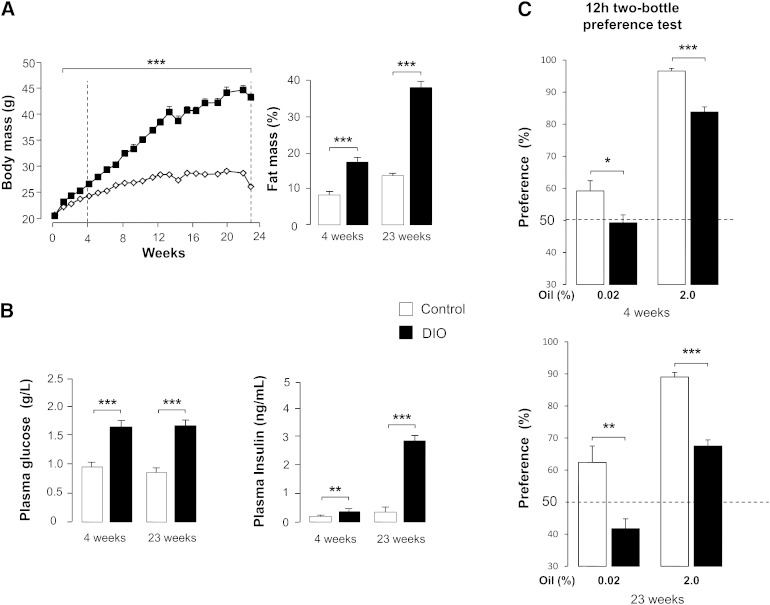
Comparison of body composition, blood parameters, and preference for lipids in controls and mice subjected to DIO for 4 and 23 weeks. A: Evolution of body and fat mass in controls and DIO mice. Means ± SEM, (n = 40). *P* < 0.001 (***). B: Plasma glucose and insulin levels in overnight fasted controls and DIO mice. Means ± SEM (n = 20). *P* < 0.01(**) and *P* < 0.001 (***). C: Long-term (12 h) two-bottle preference tests performed in controls and DIO mice. Animals were simultaneously subjected for 12 h to a control solution (0.3% xanthan gum in water, w/v) and to a test solution containing rapeseed oil (0.02 or 2.0%, w/v) in the control solution. Xanthan gum was used to minimize textural cues and to emulsify rapeseed oil. Dotted line represents the absence of preference. Means ± SEM (n = 10–12). *P* < 0.05 (*), *P* < 0.01 (**), and *P* < 0.001 (***).

To explore whether the preference for lipid-rich foods might be affected by eating habits leading to obesity, mice were chronically fed obesogenic diets (Table 1). As expected, animals chronically subjected to the HF diet rapidly developed obesity (**Fig. 1A**). A 2- and 3-fold increase in fat mass was found after 4 and 23 weeks of diet, respectively. Fasted plasma glucose levels and insulinemia were also enhanced in DIO mice, suggesting the development of insulin resistance at only 4 weeks of treatment (Fig. 1B). Long-term (12 h) two-bottle preference tests performed in control mice subjected to different concentrations of rapeseed oil (0.01–2.0% w/v) established that animals were able to detect and prefer oily solution when lipid concentration reached 0.02% in emulsion, the maximal preference being observed with 2.0% oil (13). In contrast to controls, DIO mice were unable to properly detect a low concentration of oil (0.02%) whatever the duration of the treatment (4 and 23 weeks). Moreover, mice fed a HF diet for 4 or 23 weeks showed a lower attraction for 2.0% oily solution than controls (Fig. 1C). Altogether, these data indicate that the chronic consumption of HF diet increases the detection threshold (decreased sensitivity) for lipids. Similar results were obtained when mice were chronically fed another obesogenic diet [high–fat/high-sucrose (HFHS) diet, supplementary data I], suggesting that it is the induction of obesity, rather than the qualitative diet composition, that might be the major determinant of this behavioral change.

### Preference for lipids is inversely correlated with the fat mass in the mouse

**Fig. 2.**
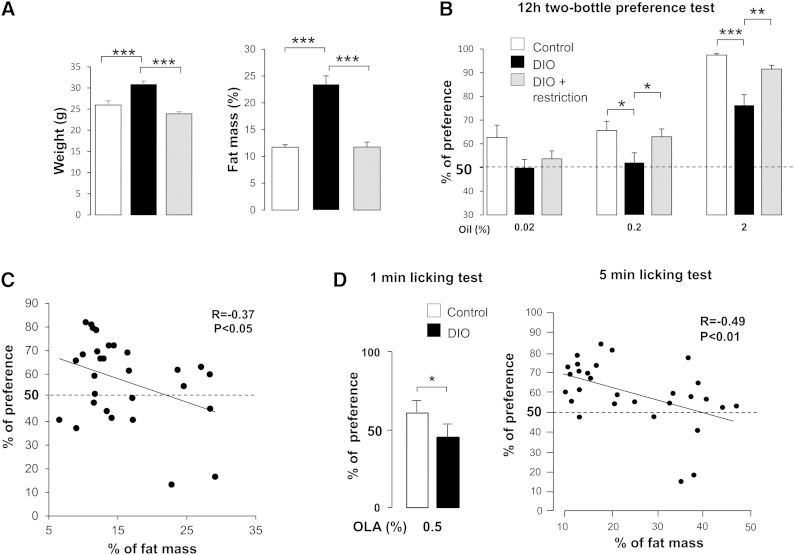
Effects of fat mass on the preference for lipids in the mouse. A: Evolution of body mass and fat mass in controls, DIO, and energy-restricted DIO mice. Body fat mass was determined at 8 weeks using a quantitative echo-MRI. Means ± SEM (n = 10). *P* < 0.001 (***). B: Long-term (12 h) two-bottle preference tests performed after 8 weeks of experiment in control, DIO, and energy-restricted DIO mice. Animals were simultaneously subjected, for 12 h, to a control solution (0.3% xanthan gum in water, w/v) and a test solution containing rapeseed oil (0.02, 0.2, or 2.0%, w/v) in the control solution. Xanthan gum was used to minimize textural cues and to emulsify rapeseed oil. Dotted line represents the absence of preference. Means ± SEM (n = 9–10), *P* < 0.05 (*), *P* < 0.01 (**), and *P* < 0.001 (***). C: Correlation between fat mass (in percent of body mass) and preference for rapeseed oil (%) determined by using the two-bottle preference tests performed in controls, DIO, and energy-restricted DIO mice. Dotted line represents the absence of preference. D: Short-term (1 min) licking tests in controls and DIO (HFHS diet) mice. Animals were subjected successively in a randomized manner to a control solution (mineral oil) or to 0.5% OLA in the control solution. Dotted line represents the absence of taste preference. Means ± SEM (n = 7–6), *P* < 0.05 (*). A correlation between the fat mass (in percent body mass) and the preference for OLA (%) determined using licking tests is also represented. Dotted line represents the absence of preference.

**Fig. 3.**
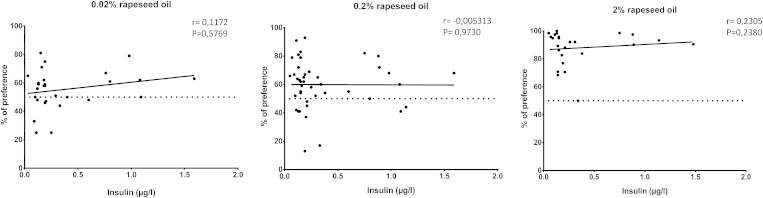
Correlation between plasma insulin levels and preference for fat. Experiments were performed in controls, DIO, and energy-restricted DIO mice subjected to two-bottle preference tests in the presence of various concentrations of rapeseed oil. Dotted line represents the absence of preference. Plasma insulin levels were assayed using a commercial kit.

To explore whether this relative orosensory indifference to dietary lipids was related to the fat mass, mice fed the HF diet ad libitum for 4 weeks were then subjected to a caloric restriction [i.e., 60% (w/w) of the previous HF diet consumption] until their fat mass returned to control values (**Fig. 2A**). Comparative analysis of preference tests performed during the weight gain/weight loss sequence revealed that the preference for lipids was tightly related to fat mass in the mouse. Indeed, mice fed the calorie-restricted diet displayed a behavior similar to that of controls maintained on regular chow, with a greater preference for 0.2% and 2.0% oil than that found when they were obese (Fig. 2B). A significant inverse correlation between attraction for fat and adiposity was found (Fig. 2C). A similar association was also found in HFHS-fed mice subjected to the licking paradigm. Indeed, the preference for OLA, determined using brief (5 min) computer-controlled licking tests and the fat mass accumulation assayed by echo-MRI, supports the fact that orosensory detection of lipids is dependent on adipose tissue size (Fig. 2D). Although most taste bud cells express insulin receptor (20), obesity-associated insulin resistance does not play a role in the preference for oily solution. Indeed, no correlation between plasma insulin levels and fat preference was found in controls, DIO, or DIO-restricted mice (**Fig. 3**).

### Lipid-mediated regulation of CD36 protein levels in gustatory papillae is impaired by DIO

**Fig. 4.**
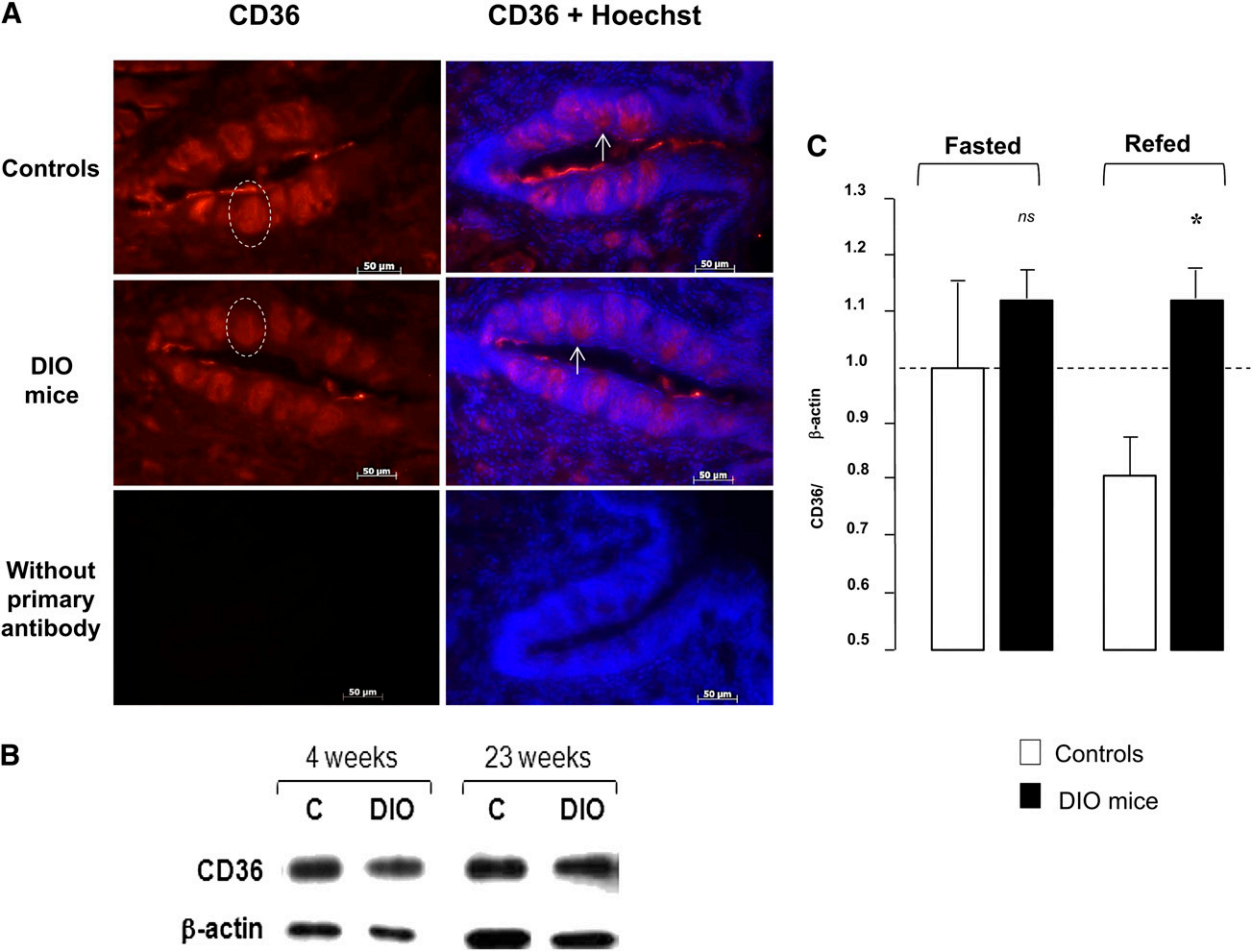
Effect of DIO on the lipid-mediated regulation of CD36 protein levels in gustatory papillae in mice. A: Immunohistochemistry analysis of CD36 expression in CVP from overnight-fasted controls and DIO mice subjected to the HF diet for 4 weeks. Dotted circles show one of the taste buds in CVP. Arrows point to examples of apical location of CD36 in taste buds. No fluorescent staining was observed when the primary antibody was omitted. B: Relative CD36 protein levels determined by Western blotting in CVP from overnight-fasted controls and DIO mice subjected to the HF diet for 4 and 23 weeks. Each point corresponds to a pool of total proteins from three mice CVP. C: Relative CD36 protein levels determined by Western blotting in CVP from controls and DIO (HF diet for 4 weeks) mice fasted overnight or refed ad libitum the HF diet for 1 h. Each point corresponds to a pool of total proteins from five mice CVP. Means ± SEM (n = 3), *P* < 0.1 (*). ns, nonsignificant.

In gustatory mucosa, CD36 is thought to be a lipid receptor implicated in the orosensory detection of dietary lipids (i.e., LCFA). To determine whether the lower lipid preference found in obese mice was related to lingual CD36 protein, comparison of immunolocalization of CD36 was undertaken in CVP from overnight-fasted controls and DIO mice. As shown in **Fig. 4A**, CD36 displays the same expression pattern in obese as in control mice. To confirm this observation, Western blotting analysis was performed in CVP. Similar expression levels of CD36 were found in control and obese mice, whatever the duration of HF diet (Fig. 4B) or the composition of obesogenic diet used (HFHS - supplementary data II). It was recently reported in mouse CVP that CD36 protein levels are downregulated by lipids during the postprandial period (11), and a disturbance in this regulation affects the spontaneous attraction for fat (13). Therefore, it was tempting to speculate that the lower attractive effect of oily solutions found in obese mice might be due to a dysfunction in this regulatory loop controlling the amounts of CD36 in CVP during a meal. To explore this hypothesis, CD36 protein levels in CVP were compared in fasted and refed controls and DIO mice. Because CD36 in CVP is lipid sensitive and its postprandial decrease is significant 60 min after the beginning of a meal (11), animals were refed the HF diet for 1 h. Consistent with data shown in Fig. 4B, fasted controls and DIO mice displayed similar CD36 expression levels in CVP. By contrast, the drop in CD36 content of CVP found 1 h after refeeding in controls was not retrieved in obese mice (Fig. 4C).

### Obesity disturbs the calcium signaling in CD36-positive gustatory cells from CVP

**Fig. 5.**
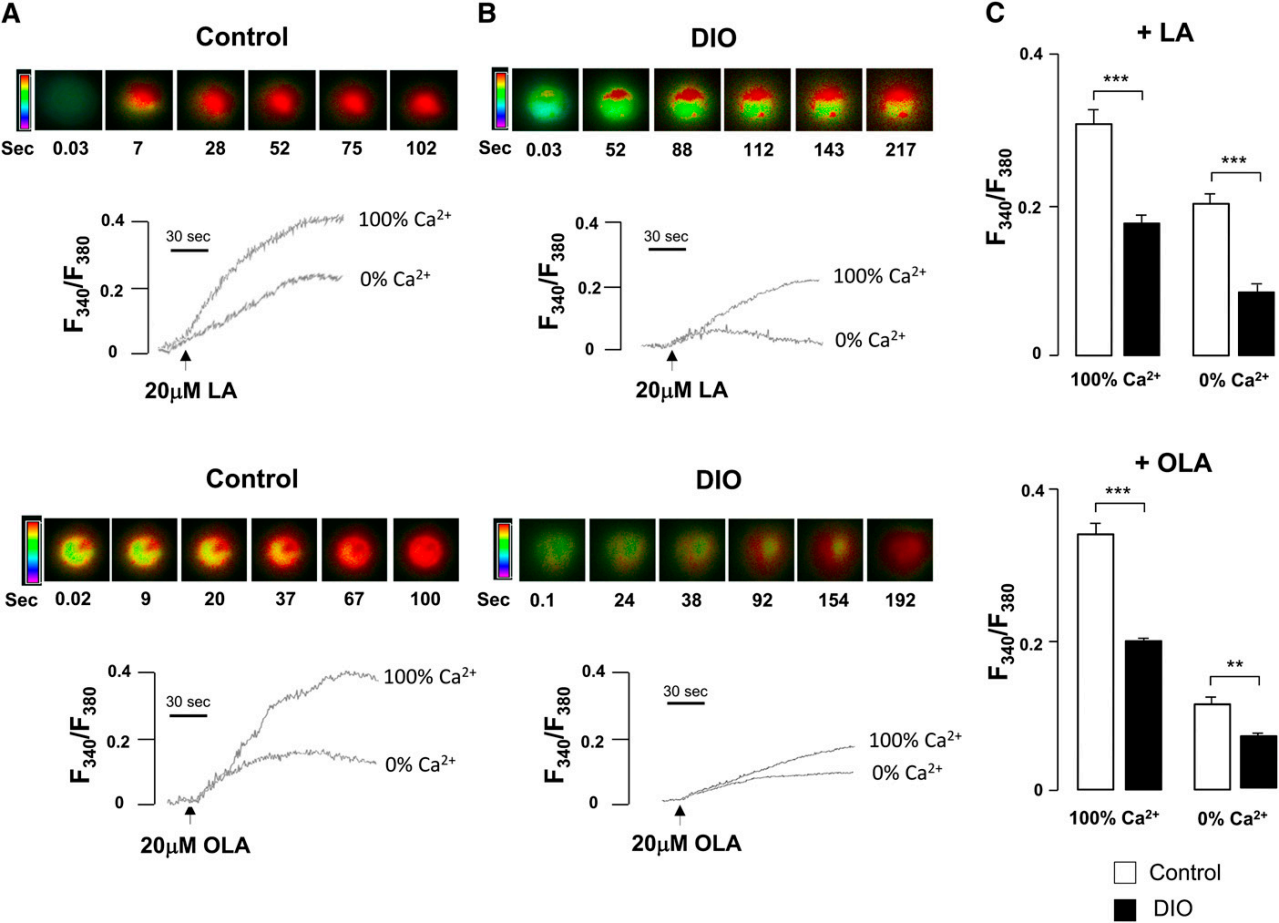
Effects of DIO on the lipid-mediated regulation of the calcium signaling in CD36-positive taste bud cells. Ca^2+^ imaging studies were performed in Ca^2+^-free (0% Ca^2+^) or in calcium-containing (100% Ca^2+^) media. The changes in intracellular Ca^2+^ (F340/F380) were monitored under the Nikon microscope (TiU) by using S-fluor 40× oil immersion objectives, as described in the Materials and Methods section. Colored time-lapse changes show the kinetics of the rise in [Ca^2+^]_i_ levels in a CD36-positive taste bud cell freshly isolated from CVP from controls and DIO (HF for 4 weeks) mice following addition of 20 μM LA or OLA in the medium. The arrows indicate when LA or OLA was added into the cuvette without interruptions in the recording. Representative data found in controls (A) and DIO mice (B). C: Bar graphs showing the changes in [Ca^2+^]_i_ obtained by pooling data from three separate experiments. Means ± SEM, (n = 7), *P* < 0.01 (**) and *P* < 0.001 (***).

In mouse CVP, LCFA-mediated activation of CD36 triggers a complex signaling cascade, producing a huge rise in intracellular free calcium concentrations ([Ca^2+^]_i_) at the origin of neurotransmitters release (8–10). To assess whether obesity-mediated decrease in fat preference is due to a dysfunction in the CD36-dependent signaling, calcium imaging experiments were performed on freshly purified CD36-positive taste bud cells from control or from mice fed the HF for 4 weeks. The effects of the two main LCFAs present in rapeseed oil, i.e., linoleic acid (LA) and OLA, were studied. Consistent with previously published data (8, 10), addition of LA or OLA led to a rapid rise in [Ca^2+^]_i_ in taste bud cells from control mice (**Fig. 5A**). LCFA-evoked rise in [Ca^2+^]_i_ in a medium containing calcium (100% Ca^2+^) was found to be decreased, albeit to a lesser extent, in the absence of calcium (0% Ca^2+^), suggesting that LCFA led to the recruitment of calcium from both extra- and intracellular pools (Fig. 5A). Although taste bud cells isolated from CVP of obese mice were responsive to LA or OLA (Fig. 5B), [Ca^2+^]_i_ rises were dramatically lower than those found in CVP from lean controls in 0 or 100% calcium media (Fig. 5C).

## DISCUSSION

Obesity is one of the major public health challenges in the world by reason of the deleterious effects of its associated diseases (i.e., type 2 diabetes, vascular disorders, hypertension, cancer). Although the origin of the obesity epidemic is clearly multi-factorial, eating habits, especially overconsumption of high-palatable energy-dense foods, are thought to play a significant role in this situation (4). Several lines of evidence indicate that laboratory rodents and humans display a preference for lipid-rich foods through poorly known mechanisms (21, 22). However, whether preference for fat is the cause and/or the consequence of obesity remains to be elucidated. The present report brings the first demonstration that DIO alters the gustatory pathway involved in the detection of dietary lipids in the mouse. Obese mice not only are unable to properly detect low concentrations of oil (0.02%), but also display a weaker preference for high concentrations (2.0% oil) than do controls, demonstrating that obesogenic diets enhance the oral detection threshold (i.e., decrease the sensitivity) for lipids. It is noteworthy that the composition of the energy-dense diets used, especially the addition of sucrose, does not seem to play a significant role in this behavioral change, because the two obesogenic diets used have produced similar results (Fig. 1 and SD 1).

A similar rightward shift in the dose response to oil was also observed in obesity-altering central reward functions in DIO rats (23). Our data strongly suggest that obesity also disturbs the function of the peripheral orosensory system, because a lower preference for lipids is reproduced in obese mice by the use of computer-controlled licking tests in conditions minimizing postingestive influences (1–5 min). Although oral lipase activity is especially efficient in the mouse (24) and is involved in the detection threshold of lipids in human (16), a decrease in the expression and/or activity of lingual lipase in obese mice seems unlikely. Indeed, DIO animals also displayed a low attraction for solutions containing a free FA (OLA). Our data support the fact that the size of fat mass is a major determinant for the modulation of oral fat sensitivity. Indeed, a reversal of obesity by a chronic food restriction corrects the detection thresholds and preference for oily solutions. Moreover, lipid preference (explored by using short-term licking tests) is inversely correlated to fat mass (assayed by echo-MRI) (Fig. 2). The disturbance in the attraction for lipids found in DIO mice appears to be independent of obesity-associated insulin resistance because a correlation between preference for fat and plasma insulin levels is lacking (Fig. 3).

These findings prompted us to explore the mechanism responsible for the obesity-mediated decrease in oral fat detection. CD36 expression in CVP from lean and obese mice was investigated. Indeed, lingual CD36 is known to play a major role in the preference for fat. Furthermore, CD36 expression is lipid-sensitive in mouse CVP (11). Finally, a decrease in CD36 expression level was recently reported in the gustatory mucosa of DIO rats (25). We did not observe such a change in fasted DIO mice regardless of the obesogenic diets used (Fig. 4 and SD 2). This discrepancy might be due to experimental differences (e.g., duration of treatment, composition of diets) that might lead to a different inflammatory status. Indeed, inflammation interferes with taste cell renewal (26). The fact that the number of taste buds is clearly decreased in CVP from obese rats (25), in contrast to that found in obese mice (Fig. 4A), is consistent with this assumption. By contrast, we have found that the dynamic downregulation of lingual CD36 protein levels, previously observed during the refeeding of fasted lean mice (11, 13), was not retrieved in obese animals. A similar CD36 dysfunction in CVP had already been identified in GLP-1R-null mice in which attraction for oily solutions was also reduced (13). Direct evidence of a differential change in the cell surface expression of CD36 in CVP from lean and obese mice seems unrealistic by reason of the scarcity of the biological material (1 CVP per mouse, containing a few dozen taste buds constituted of a few dozen CD36-expressing cells). The recent development of a human fungiform taste cell line could be a good alternative approach to get more information on this aspect in the future (27). Altogether our data strongly suggest that the lack of preference of obese mice for fat might be due to alterations in the lipid-sensing system related to the lingual CD36. Consistent with this assumption, a reduction of the LCFA-mediated Ca^2+^ mobilization was observed using calcium imaging in CD36-positive taste bud cells freshly isolated from DIO mice, as compared with their lean counterparts (Fig. 5). This finding brings the first demonstration that obesity affects the lipid-mediated calcium signaling in taste bud cells. Although CD36 and GPR120 may be coexpressed in taste bud cells (13), it is tempting to speculate that the lower calcium response to LCFA found in CVP of obese mice is mainly dependent on CD36. Indeed, calcium response to LA was deeply reduced when taste bud cells were previously treated with the specific and irreversible CD36 binding inhibitor sulfo-*N*-succinimidyl-oleate or in CD36-negative TBC (10).

**Fig. 6.**
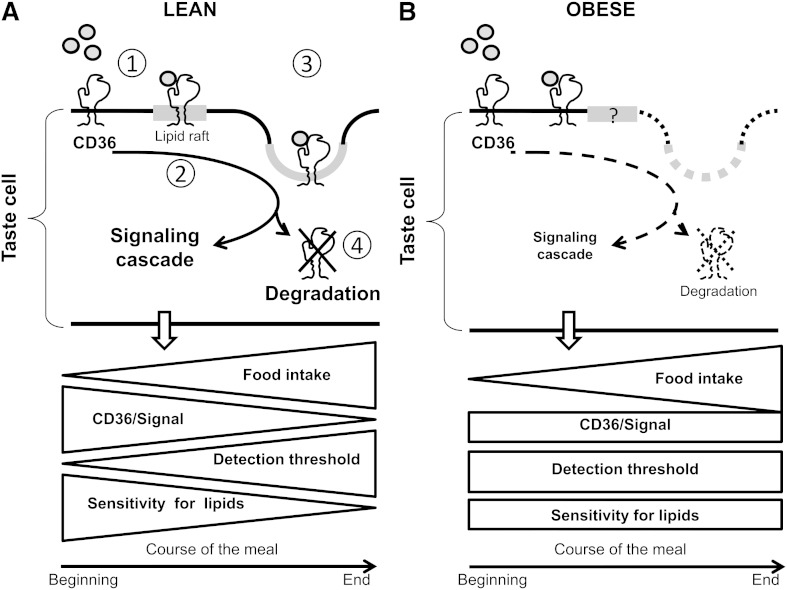
Working hypothesis. A: The following scenario might explain the relationship between lingual CD36 and preference for lipids in control animals. At the beginning of a meal, the LCFA/CD36 complex in the lipid rafts (1) activates the lipid-dependent signaling cascade in taste bud cells (2). This event is followed by a progressive endocytosis (3) and the degradation of CD36 by the ubiquitin/proteasome pathway (4). This downregulation is responsible for a gradual enhancement of the orosensory detection threshold of lipids, leading to a progressive decrease of the preference for fat during a meal. B: Obesity might impair this regulatory cascade by limiting the amount of CD36 in lipid rafts and thus, the subsequent signaling and degradation of CD36. In consequence, the CD36 levels in the plasma membrane and the related signaling cascade remain relatively stable during a meal in obese mice, as the lipid detection threshold and preference for fat, in contrast to what occurs in lean animals.

The following working model, reconciling the present data with those of the literature, might explain the role played by the lingual CD36 in the decreased fat preference found in DIO mice. In lean animals (**Fig. 6A**), *i*) LCFA binding to lingual CD36 might induce its translocation in lipid-rafts, mostly found in the apical side of mature taste bud cells (1, 28); *ii*) by promoting the interaction of CD36 with Src-PTK (29), this event would trigger the signaling cascade (8); *iii*) this early event, which might contribute to the attraction for fatty foods at the beginning of a meal (11), would be followed by a progressive disappearance of CD36 from the plasma membrane which might occur via a caveolae-mediated endocytotic process (28); *iv*) once in the cell, CD36 might undergo a degradation by the ubiquitin/proteasome pathway, as previously demonstrated in the small intestine (30). This CD36 downregulation might lead to a progressive decrease in the preference for fatty foods during a meal by reason of a gradual rise in the lipid detection threshold (i.e., a loss of sensitivity) (11). We postulate that obesity might impair this signaling machinery by limiting the CD36 amounts in lipid rafts, which might restrain the subsequent signaling cascade and CD36 degradation. In these conditions, plasma membrane CD36 levels, related signaling, lipid detection threshold, and preference for fat might remain relatively stable during a meal (Fig. 6B). The lower preference for lipids found in DIO mice is consistent with such a scenario. An abnormal cell surface CD36 location was also found in muscular cells derived from obese patients (31), suggesting that obesity affects the distribution of CD36 in different cell types. The origin of this dysfunction remains to be established. A decrease in plasma membrane fluidity due to chronic overconsumption of saturated fat, endocrine disturbances (e.g., insulin resistance, hyperleptinemia, drop in the plasma GLP-1 levels), and/or inflammation related to obesity are plausible candidates. Consistent with this idea, insulin, leptin, and GLP-1 receptors are found in TBC (20, 32, 33). Moreover, plasma membrane localization of CD36 is controlled by insulin in myocytes (34), whereas leptin acts as a modulator of sweet taste (32), and fat taste sensitivity is controlled by GLP-1 (13). Finally, it has recently been reported that a lipopolysaccharide-induced inflammation disturbs the physiology of gustatory papillae in the mouse (26).

Our results provide the first evidence that obesity may impair the orosensory detection of free LCFA via a mechanism in which the lingual CD36 plays a role in the mouse. This phenomenon seems to be tightly linked to the size of the fat mass. Because CD36 is also expressed in human taste buds (35), and because induced weight loss by bariatric surgery improves taste acuity, rending energy-dense foods less pleasant (36), such a dysregulation might also exist in humans. Given that the peripheral gustatory system potentially influences food choice and, thus, eating behavior (37), a better understanding of the regulatory loops controlling the function of lipid receptors expressed in taste buds might open novel avenues in the pharmacological and/or nutritional treatment of obesity.

## Supplementary Material

Supplemental Data
