# Supplementary material for: Obesity alters the gustatory perception of lipids in the mouse: plausible involvement of lingual CD36
Source: J Lipid Res. 2013 Sep;54(9):2485–94. doi: 10.1194/jlr.M039446 (PMC3735945; doi:10.1194/jlr.M039446)
Supplement: Supplemental Data [file supp_54_9_2485__index.html]

Obesity alters the gustatory perception of lipids in the mouse: plausible involvement of the lingual CD36 — Obesity alters the gustatory perception of lipids in the mouse: plausible involvement of lingual CD36 — Supplemental Data 

# Obesity alters the gustatory perception of lipids in the mouse: plausible involvement of lingual CD36

## 

**Files in this Data Supplement:**

- Supplemental Figure 1 - Comparison of body composition, plasma insulin and preference for lipids in controls and mice chronically subjected to a High Fat High Sucrose- (HFHS) diet.
- Supplemental Figure 2 - Effects of a High Fat-High Sucrose (HFHS) diet on the CD36 protein levels in gustatory papillae in mice
