# Supplementary material for: Obesity alters the gustatory perception of lipids in the mouse: plausible involvement of lingual CD36
Source: J Lipid Res. 2013 Sep;54(9):2485–94. doi: 10.1194/jlr.M039446 (PMC3735945; doi:10.1194/jlr.M039446)
Supplement: Supplemental Data [file supp_M039446_jlr.M039446-1.pdf]

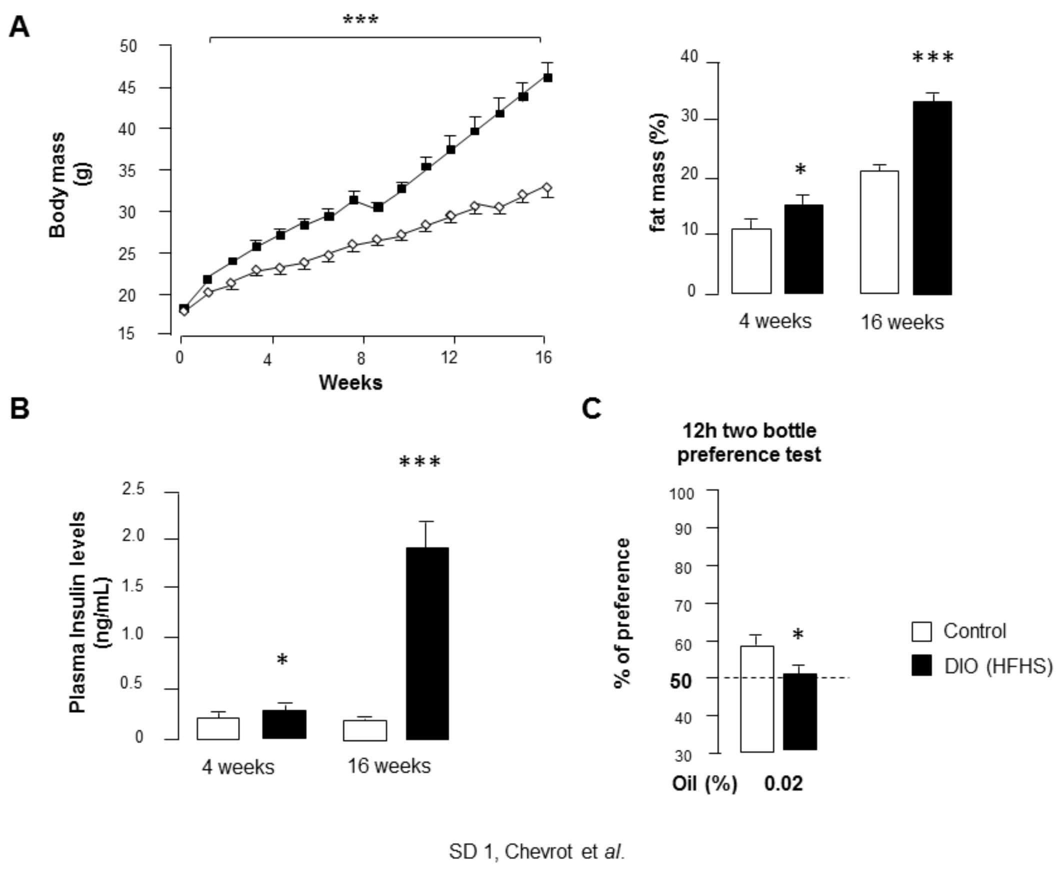

SD Fig.I: ***Comparison of body composition, plasma insulin and preference for lipids in controls and mice chronically subjected to a High Fat High Sucrose- (HFHS) diet.***

A- Evolution of body and fat mass in controls and obese mice. Means ± SEM, (n=20). P<0.001 (***).

B- Plasma insulin levels in overnight fasted controls and obese mice. Means ±SEM (n=20). P<0.05 (*) and P<0.001 (***).

C- Long-term (12 h) two-bottle preference tests performed in control and obese mice. Animals were simultaneously subjected for 12 h to a control solution (0.3% Xanthan gum in water, w/v) and to a test solution containing rapeseed oil (0.02%, w/v, in the control solution). Xanthan gum was used to minimize textural cues and to emulsify rapeseed oil. Dotted line represents the absence of preference. Means ± SEM (n=12), P<0.05 (*).
