# Supplementary material for: Obesity alters the gustatory perception of lipids in the mouse: plausible involvement of lingual CD36
Source: J Lipid Res. 2013 Sep;54(9):2485–94. doi: 10.1194/jlr.M039446 (PMC3735945; doi:10.1194/jlr.M039446)
Supplement: Supplemental Data [file supp_M039446_jlr.M039446-2.pdf]

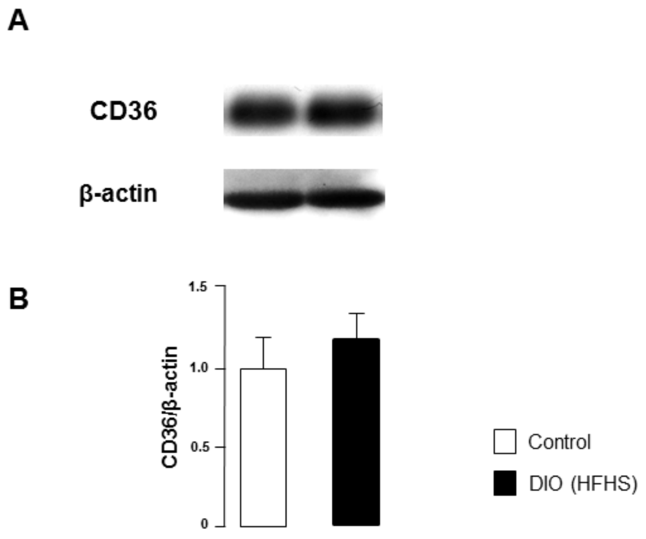

SD 2, Chevrot et *al*.

SD Fig. II: ***Effects of a High Fat-High Sucrose (HFHS) diet on the CD36 protein levels in gustatory papillae in mice***

A- Representative data of relative CD36 protein levels determined by Western blotting in circumvallate papillae (CVP) from overnight fasted control and obese mice.

B- Bar graph representation of the relative CD36 protein levels in CVP from overnight fasted mice (n=3, each point corresponds to a pool of total proteins from 3 mice CVP).
